# Supplementary material for: SABRE populates ER domains essential for cell plate maturation and cell expansion influencing cell and tissue patterning
Source: eLife. 2021 Mar 9;10:e65166. doi: 10.7554/eLife.65166 (PMC7987345; doi:10.7554/eLife.65166)
Supplement: Supplementary file 1. [file elife-65166-supp1.docx]

**Supplementary Material**

**Supplementary File 1a.** Primers used in this study and the plasmid constructs they are used to generate respectively.

| **Identifier** | **Sequence** | **Purpose** | **For Plasmid** |
| --- | --- | --- | --- |
| DC65 | caccATGGAGGTTACACCTGAC | Clone transcript |  |
| DC164 | ccatTCAGTGCGCGAGTAAGCTTC | protospacer for knockout *SABRE* | pMH-SAB-stop |
| DC165 | aaacGAAGCTTACTCGCGCACTGA |  |  |
| DC175 | GGGGACAAGTTTGTACAAAAAAGCAGGCTTAGTGATTGAGCAACAGCTATTGC | PCR amplify 5' arm for inserting stop cassette with HDR | pGEM-SAB-stop |
| DC176 | GGGGACAACTTTGTATAGAAAAGTTGGGTGGAACCCTGCTGGCTATC |  |  |
| DC177 | GGGGACAACTTTGTATAATAAAGTTGTAGCTTCCGGTTAGCTGGT | PCR amplify 3' arm for inserting stop cassette with HDR |  |
| DC168 | GGGGACCACTTTGTACAAGAAAGCTGGGTTCTGCTGGATACAGTGAGATG |  |  |
| DC265 | TGTAATTATTCCAGAAGTGTTAGG | Sequence knockout genotyping PCR |  |
| DC191 | CAAGATAACCTCCACATCCG | Sequence knockout genotyping PCR, clone transcript |  |
| DC266 | GCAGAAAGAATTGAGGTTGG | Genotype *∆sabre* knockout |  |
| DC267 | CCGATCAGAATGATCAACAAG |  |  |
| DC314 | ccatGGCCGTGACTCTCCCCTCTG | protospacer for inserting maize ubiquitin promoter or SABRE N terminal tag | pMH-SAB-N |
| DC315 | aaacCAGAGGGGAGAGTCACGGCC |  |  |
| DC316 | ccatCGATACCCCATCAGCTTACG | protospacer for inserting SABRE C terminal tag | pMH-SAB-C |
| DC317 | aaacCGTAAGCTGATGGGGTATCG |  |  |
| DC322 | GGGGACAAGTTTGTACAAAAAAGCAGGCTTACTAGGACGCTGGGCTAAG | PCR amplify 5' arm for inserting ubiquitin promoter or SABRE N terminal tag with HDR | pGEM-Ubipro-SAB |
| DC323 | GGGGACAACTTTGTATAGAAAAGTTGGGTGGAATCCAACACTTCAGAGGC |  |  |
| DC324 | GGGGACAACTTTGTATAATAAAGTTGTAATGGAGGTTACACCTGACAAAT | PCR amplify 3' arm for inserting ubiquitin promoter or SABRE N terminal tag with HDR |  |
| DC325 | GGGGACCACTTTGTACAAGAAAGCTGGGTTGAACCCTGCTGGCTATC |  |  |
| DC326 | GGGGACAAGTTTGTACAAAAAAGCAGGCTTAAGTTCAAGGATAAGTTACCCGC | PCR amplify 5' arm for inserting SABRE C terminal tag with HDR | pGEM-SAB-3mNG  pGEM-SAB-3GFP |
| DC327 | GGGGACAACTTTGTATAGAAAAGTTGGGTGATCCAAGTTCTCGTAAGCTGATG |  |  |
| DC328 | GGGGACAACTTTGTATAATAAAGTTGTACAGCAATAACCATCCAGTTTTGTA | PCR amplify 3' arm for inserting SABRE C terminal tag with HDR |  |
| DC329 | GGGGACCACTTTGTACAAGAAAGCTGGGTTGCTGTGAAACAGTGAGGTC |  |  |
| DC403 | GGTCACGTGCTTGCAT | Genotype ubiquitin promoter insertion or SABRE N terminal tag |  |
| DC404 | CGTCTTTGAGTCGTTGAAAAC |  |  |
| DC405 | ACATACATTCTGTAGCACTCAC | Genotype SABRE C terminal tag |  |
| DC406 | GAACAAGTGATTTGGTTCCTG |  |  |
| DC1059 | TTCTTGTTTCACGACAGGG |  |  |
| DC1060 | CACTCGGGATTGACATGAAC |  |  |
| DC473 | CCAAGAGGTCAGCCTTTC | Sequence SABRE C terminal tag |  |
| DC474 | GACGTGAAGGACCAAAGC |  |  |
| DC475 | GCATACGAAACAATACCGATG | Sequence ubiquitin promoter insertion |  |
| DC625 | GGGGACAACTTTTCTATACAAAGTTGTAGGATCCATGGTGAGTAAAGGCGAGG | Amplify mNeonGreen CDS | pENTR R4R3 C/Nterm BamHI 1xSc_mNeon |
| DC626 | GGGGACAACTTTATTATACAAAGTTGTTTACTTATACAATTCGTCCATACCCATC | Amplify mNeonGreen CDS with stop codon | pENTR R4R3 Cterm BamHI 1xSc_mNeon |
| DC627 | GGGGACAACTTTATTATACAAAGTTGTCTTATACAATTCGTCCATACCCATC | Amplify single mNeonGreen CDS without stop codon | pENTR R4R3 Nterm BamHI 1xSc_mNeon |
| DC632 | CAATGGTTGACGGATCA | Sequence mNeonGreen tag genotyping PCR |  |
| DC633 | TTAGAACGGCACCAATCA |  |  |
| DC634 | GGCTATGGTAGATGGCAGT |  |  |
| DC635 | CTACGGCACCAATCGGCA |  |  |
| DC791 | TAGCGTGGATCCATGGTAAGCAAAGGAGAGGAGG | Amplify mNeonGreen CDS at 2nd position for triple tag, or first position for double tag | pENTR R4R3 C/Nterm BamHI 2xPp_Sc_mNeon |
| DC792 | TAGCGTAGATCTCTTGTATAACTCATCCATGCCC |  |  |
| DC793 | TAGCGTGGATCCATGGTGAGTAAAGGCGAGG | Amplify mNeonGreen CDS at 1st position for triple tag | pENTR R4R3 C/Nterm BamHI 3xSc_Pp_Sc_mNeon |
| DC794 | TAGCGTAGATCTCTTATACAATTCGTCCATACCCATC |  |  |
| DC818 | GGGGACAACTTTTCTATACAAAGTTGGGCTAGAGATAATGAGCATTGCATGTCTAAG | Amplify maize ubiquitin promoter to make the entry clone for HDR | pENTR-R4R3-Ubiquitin-pro |
| DC819 | GGGGACAACTTTATTATACAAAGTTGTGCAGAAGTAACACCAAACAACAGG |  |  |
| DC1055 | ccatGTTGCCAAGTTCGCCGGGCT | protospacer for switch Lifeact-mRuby to mCherry-KDEL | pMH-mRuby2-2ps |
| DC1056 | aaacAGCCCGGCGAACTTGGCAAC |  |  |
| DC1057 | ccatGTCATGGAAGGTTCGGTCAA |  |  |
| DC1058 | aaacTTGACCGAACCTTCCATGAC |  |  |

**Supplementary File 1b.** Plasmids used to transform moss and the lines generated from those transformations.

| **CRISPR plasmid** | **Homology plasmid** | **Background** | **Generated line** |
| --- | --- | --- | --- |
| pMH-SAB-stop | pGEM-SAB-stop | wild type | *∆sab*/wildtype |
| pMH-SAB-stop | pGEM-SAB-stop | GFP-tubulin | *∆sab*/GFP-tubulin |
| pMH-SAB-stop | pGEM-SAB-stop | Lifeact-GFP | *∆sab*/Lifeact-GFP |
| pMH-SAB-stop | pGEM-SAB-stop | SP-GFP-KDEL | *∆sab*/ER-GFP |
| pMH-SAB-stop | pGEM-SAB-stop | NLS-GFP-GUS/SNAP-TM-mCherry | *∆sab*/NLS-GFP/SNAP-mCh |
| pMH-SAB-C | pGEM-SAB-3GFP | wild type | SAB-3GFP |
| pMH-SAB-C | pGEM-SAB-3mNG | mCherry-tubulin | SAB-3mNG/mCh-tub |
| pMH-SAB-C | pGEM-SAB-3mNG | Lifeact-mRuby | SAB-3mNG/LAmR |
| pMH-SAB-N | pGEM-Ubipro-SAB | SAB-3mNG/mCh-tub | OE-SAB-3mNG/mCh-tub |
| pMH-SAB-N | pGEM-Ubipro-SAB | SAB-3mNG/LAmR | OE-SAB-3mNG/LAmR |
| pMH-mRuby2-2ps | pTZ-SP-mCherry-KDEL | SAB-3mNG/LAmR | SAB-3mNG/ER-mCherry |
| pMH-mRuby2-2ps | pTZ-SP-mCherry-KDEL | OE-SAB-3mNG/LAmR | OE-SAB-3mNG/ER-mCherry |
| pMH-SAB-stop | pGEM-SAB-stop | OE-SAB-3mNG/ERmCh | *∆sab*/ER-mCherry |

**Supplemental File 1c.**

One Way ANOVA for Figure 1C

Analysis of Variance Results

| Source | DF | SS | MS | F | P |
| --- | --- | --- | --- | --- | --- |
| Total | 148 | 22.542134 | 0.15231172 |  |  |
| A | 3 | 11.654699 | 3.8848996 | 51.739499 | < .0001 |
| Error | 145 | 10.887435 | 0.07508576 |  |  |

| Tukey's All Pairs Comparison | | | | |
| --- | --- | --- | --- | --- |
| Comparison | Mean Difference | \|q\| | P | 95% CL |
| Wild type vs ∆sab/GFP-tub | 0.674116 | 12.8543 | < .0001 | 0.48136 to 0.86688 |
| Wild type vs ∆sab/wt | 0.626835 | 12.2276 | < .0001 | 0.43841 to 0.81526 |
| Wild type vs GFP-Tub | 0.120015 | 2.221 | 0.3987 | -0.078606 to 0.31864 |
| GFP-Tub vs ∆sab/GFP-tub | 0.554101 | 12.6589 | < .0001 | 0.39321 to 0.71499 |
| GFP-Tub vs ∆sab/wt | 0.50682 | 11.9668 | < .0001 | 0.35115 to 0.66249 |
| ∆sab/wt vs ∆sab/GFP-tub | 0.047281 | 1.1733 | 0.8403 | -0.10084 to 0.1954 |

**Supplemental File 1d.**

One Way ANOVA for Figure 2E

Analysis of Variance Results

| Source | DF | SS | MS | F | P |
| --- | --- | --- | --- | --- | --- |
| Total | 231 | 1.12733e+08 | 488021.64 |  |  |
| A | 3 | 65689678 | 21896559 | 106.12379 | < .0001 |
| Error | 228 | 47043322 | 206330.36 |  |  |

| Tukey's All Pairs Comparison | | | | |
| --- | --- | --- | --- | --- |
| Comparison | Mean Difference | \|q\| | P | 95% CL |
| WT base vs *∆sabre* tip | 1761.79 | 22.2642 | < .0001 | 1472.2 to 2051.4 |
| WT base vs WT tip | 1334.01 | 14.7156 | < .0001 | 1002.2 to 1665.8 |
| WT base vs *∆sabre* base | 910.541 | 10.575 | < .0001 | 595.4 to 1225.7 |
| *∆sabre* base vs *∆sabre* tip | 851.251 | 16.0416 | < .0001 | 657.03 to 1045.5 |
| *∆sabre* base vs WT tip | 423.472 | 6.1301 | 0.0001 | 170.63 to 676.32 |
| WT tip vs *∆sabre* tip | 427.778 | 7.1097 | < .0001 | 207.56 to 648 |

**Supplemental File 1e.**

One Way ANOVA for Figure 2F.

Analysis of Variance Results

| Source | DF | SS | MS | F | P |
| --- | --- | --- | --- | --- | --- |
| Total | 231 | 413.58583 | 1.7904148 |  |  |
| A | 3 | 108.1622 | 36.054066 | 26.914509 | < .0001 |
| Error | 228 | 305.42363 | 1.3395773 |  |  |

| Tukey's All Pairs Comparison | | | | |
| --- | --- | --- | --- | --- |
| Comparison | Mean Difference | \|q\| | P | 95% CL |
| WT base vs *∆sabre* tip | 2.24105 | 11.1148 | < .0001 | 1.5031 to 2.979 |
| WT base vs *∆sabre* base | 1.49257 | 6.8032 | < .0001 | 0.68957 to 2.2956 |
| WT base vs WT tip | 1.06931 | 4.6293 | 0.0067 | 0.22388 to 1.9147 |
| WT tip vs *∆sabre* tip | 1.17174 | 7.6429 | < .0001 | 0.61061 to 1.7329 |
| WT tip vs *∆sabre* base | 0.423253 | 2.4046 | 0.3259 | -0.221 to 1.0675 |
| *∆sabre* base vs *∆sabre* tip | 0.748485 | 5.5357 | 0.0007 | 0.2536 to 1.2434 |

**Supplemental File 1f.**

One Way ANOVA for Figure 4E.

Analysis of Variance Results

| Source | DF | SS | MS | F | P |
| --- | --- | --- | --- | --- | --- |
| Total | 119 | 4.4267577 | 0.03719965 |  |  |
| A | 11 | 3.0277591 | 0.27525083 | 21.248835 | < .0001 |
| Error | 108 | 1.3989986 | 0.01295369 |  |  |

| Tukey's All Pairs Comparison | | | | |
| --- | --- | --- | --- | --- |
| Comparison | Mean Difference | \|q\| | P | 95% CL |
| (OE) ER-mCherry vs mCherry-Tub | 0.552608 | 14.7775 | < .0001 | 0.37592 to 0.72929 |
| (OE) ER-mCherry vs mCherry-Tub flip | 0.50407 | 13.4795 | < .0001 | 0.32739 to 0.68076 |
| (OE) ER-mCherry vs LA-mRuby flip | 0.442595 | 11.3745 | < .0001 | 0.25875 to 0.62644 |
| (OE) ER-mCherry vs LA-mRuby | 0.419826 | 10.7894 | < .0001 | 0.23598 to 0.60367 |
| (OE) ER-mCherry vs (OE) mCherry-Tub | 0.417751 | 11.5488 | < .0001 | 0.24684 to 0.58866 |
| (OE) ER-mCherry vs (OE) LA-mRuby flip | 0.415489 | 12.368 | < .0001 | 0.25677 to 0.57421 |
| (OE) ER-mCherry vs (OE) mCherry-Tub flip | 0.399192 | 11.0358 | < .0001 | 0.22828 to 0.5701 |
| (OE) ER-mCherry vs ER-mCherry flip | 0.365586 | 11.0884 | < .0001 | 0.20981 to 0.52136 |
| (OE) ER-mCherry vs (OE) ER-mCherry flip | 0.357423 | 10.4155 | < .0001 | 0.19529 to 0.51956 |
| (OE) ER-mCherry vs (OE) LA-mRuby | 0.355384 | 10.5789 | < .0001 | 0.19666 to 0.51411 |
| (OE) ER-mCherry vs ER-mCherry | 0.0780706 | 2.3679 | 0.8757 | -0.077706 to 0.23385 |
| ER-mCherry vs mCherry-Tub | 0.474537 | 13.1219 | < .0001 | 0.30367 to 0.6454 |
| ER-mCherry vs mCherry-Tub flip | 0.426 | 11.7797 | < .0001 | 0.25513 to 0.59687 |
| ER-mCherry vs LA-mRuby flip | 0.364524 | 9.6616 | < .0001 | 0.18626 to 0.54279 |
| ER-mCherry vs LA-mRuby | 0.341756 | 9.0582 | < .0001 | 0.16349 to 0.52002 |
| ER-mCherry vs (OE) mCherry-Tub | 0.339681 | 9.7335 | < .0001 | 0.1748 to 0.50457 |
| ER-mCherry vs (OE) LA-mRuby flip | 0.337418 | 10.4732 | < .0001 | 0.1852 to 0.48964 |
| ER-mCherry vs (OE) mCherry-Tub flip | 0.321122 | 9.2017 | < .0001 | 0.15624 to 0.48601 |
| ER-mCherry vs ER-mCherry flip | 0.287515 | 9.1083 | < .0001 | 0.13837 to 0.43666 |
| ER-mCherry vs (OE) ER-mCherry flip | 0.279352 | 8.4729 | < .0001 | 0.12358 to 0.43513 |
| ER-mCherry vs (OE) LA-mRuby | 0.277313 | 8.6076 | < .0001 | 0.12509 to 0.42953 |
| (OE) LA-mRuby vs mCherry-Tub | 0.197224 | 5.3691 | 0.0124 | 0.023666 to 0.37078 |
| (OE) LA-mRuby vs mCherry-Tub flip | 0.148686 | 4.0477 | 0.1699 | -0.024871 to 0.32224 |
| (OE) LA-mRuby vs LA-mRuby flip | 0.0872107 | 2.2785 | 0.9013 | -0.093632 to 0.26805 |
| (OE) LA-mRuby vs LA-mRuby | 0.0644421 | 1.6836 | 0.9887 | -0.1164 to 0.24528 |
| (OE) LA-mRuby vs (OE) mCherry-Tub | 0.0623672 | 1.7574 | 0.9841 | -0.10531 to 0.23004 |
| (OE) LA-mRuby vs (OE) LA-mRuby flip | 0.060105 | 1.8294 | 0.9783 | -0.095129 to 0.21534 |
| (OE) LA-mRuby vs (OE) mCherry-Tub flip | 0.0438083 | 1.2345 | 0.9992 | -0.12386 to 0.21148 |
| (OE) LA-mRuby vs ER-mCherry flip | 0.0102019 | 0.3167 | 1 | -0.14202 to 0.16242 |
| (OE) LA-mRuby vs (OE) ER-mCherry flip | 0.00203864 | 0.0607 | 1 | -0.15668 to 0.16076 |
| (OE) ER-mCherry flip vs mCherry-Tub | 0.195185 | 5.2195 | 0.0174 | 0.0185 to 0.37187 |
| (OE) ER-mCherry flip vs mCherry-Tub flip | 0.146648 | 3.9216 | 0.2067 | -0.030037 to 0.32333 |
| (OE) ER-mCherry flip vs LA-mRuby flip | 0.0851721 | 2.1889 | 0.9233 | -0.098674 to 0.26902 |
| (OE) ER-mCherry flip vs LA-mRuby | 0.0624035 | 1.6037 | 0.9924 | -0.12144 to 0.24625 |
| (OE) ER-mCherry flip vs (OE) mCherry-Tub | 0.0603286 | 1.6678 | 0.9895 | -0.11058 to 0.23124 |
| (OE) ER-mCherry flip vs (OE) LA-mRuby flip | 0.0580664 | 1.7285 | 0.986 | -0.10066 to 0.21679 |
| (OE) ER-mCherry flip vs (OE) mCherry-Tub flip | 0.0417697 | 1.1547 | 0.9996 | -0.12914 to 0.21268 |
| (OE) ER-mCherry flip vs ER-mCherry flip | 0.00816328 | 0.2476 | 1 | -0.14761 to 0.16394 |
| ER-mCherry flip vs mCherry-Tub | 0.187022 | 5.1715 | 0.0194 | 0.016155 to 0.35789 |
| ER-mCherry flip vs mCherry-Tub flip | 0.138484 | 3.8294 | 0.2369 | -0.032382 to 0.30935 |
| ER-mCherry flip vs LA-mRuby flip | 0.0770088 | 2.0411 | 0.9519 | -0.10125 to 0.25527 |
| ER-mCherry flip vs LA-mRuby | 0.0542402 | 1.4376 | 0.997 | -0.12402 to 0.2325 |
| ER-mCherry flip vs (OE) mCherry-Tub | 0.0521653 | 1.4948 | 0.9958 | -0.11272 to 0.21705 |
| ER-mCherry flip vs (OE) LA-mRuby flip | 0.0499031 | 1.549 | 0.9943 | -0.10232 to 0.20212 |
| ER-mCherry flip vs (OE) mCherry-Tub flip | 0.0336064 | 0.963 | 0.9999 | -0.13128 to 0.19849 |
| (OE) mCherry-Tub flip vs mCherry-Tub | 0.153415 | 3.9231 | 0.2062 | -0.03135 to 0.33818 |
| (OE) mCherry-Tub flip vs mCherry-Tub flip | 0.104878 | 2.6819 | 0.7588 | -0.079888 to 0.28964 |
| (OE) mCherry-Tub flip vs LA-mRuby flip | 0.0434024 | 1.0701 | 0.9998 | -0.14822 to 0.23503 |
| (OE) mCherry-Tub flip vs LA-mRuby | 0.0206338 | 0.5088 | 1 | -0.17099 to 0.21226 |
| (OE) mCherry-Tub flip vs (OE) mCherry-Tub | 0.0185589 | 0.4892 | 1 | -0.16069 to 0.19781 |
| (OE) mCherry-Tub flip vs (OE) LA-mRuby flip | 0.0162967 | 0.4592 | 1 | -0.15138 to 0.18397 |
| (OE) LA-mRuby flip vs mCherry-Tub | 0.137119 | 3.7328 | 0.2715 | -0.036439 to 0.31068 |
| (OE) LA-mRuby flip vs mCherry-Tub flip | 0.0885813 | 2.4115 | 0.862 | -0.084976 to 0.26214 |
| (OE) LA-mRuby flip vs LA-mRuby flip | 0.0271057 | 0.7082 | 1 | -0.15374 to 0.20795 |
| (OE) LA-mRuby flip vs LA-mRuby | 0.00433714 | 0.1133 | 1 | -0.17651 to 0.18518 |
| (OE) LA-mRuby flip vs (OE) mCherry-Tub | 0.00226222 | 0.0637 | 1 | -0.16541 to 0.16993 |
| (OE) mCherry-Tub vs mCherry-Tub | 0.134857 | 3.4485 | 0.3901 | -0.049909 to 0.31962 |
| (OE) mCherry-Tub vs mCherry-Tub flip | 0.086319 | 2.2073 | 0.9191 | -0.098447 to 0.27108 |
| (OE) mCherry-Tub vs LA-mRuby flip | 0.0248435 | 0.6125 | 1 | -0.16678 to 0.21647 |
| (OE) mCherry-Tub vs LA-mRuby | 0.00207492 | 0.0512 | 1 | -0.18955 to 0.1937 |
| LA-mRuby vs mCherry-Tub | 0.132782 | 3.1879 | 0.5151 | -0.064014 to 0.32958 |
| LA-mRuby vs mCherry-Tub flip | 0.0842441 | 2.0226 | 0.9549 | -0.11255 to 0.28104 |
| LA-mRuby vs LA-mRuby flip | 0.0227686 | 0.5293 | 1 | -0.18048 to 0.22602 |
| LA-mRuby flip vs mCherry-Tub | 0.110013 | 2.6413 | 0.7761 | -0.086782 to 0.30681 |
| LA-mRuby flip vs mCherry-Tub flip | 0.0614755 | 1.4759 | 0.9962 | -0.13532 to 0.25827 |
| mCherry-Tub flip vs mCherry-Tub | 0.0485375 | 1.2062 | 0.9994 | -0.14159 to 0.23866 |

**Supplemental File 1g.**

One Way ANOVA for Figure 4 – figure supplement 2A

Analysis of Variance Results

| Source | DF | SS | MS | F | P |
| --- | --- | --- | --- | --- | --- |
| Total | 228 | 27.536436 | 0.12077384 |  |  |
| A | 5 | 1.4323106 | 0.28646212 | 2.4471632 | 0.0349 |
| Error | 223 | 26.104125 | 0.11705886 |  |  |

| Tukey's All Pairs Comparison | | | | |
| --- | --- | --- | --- | --- |
| Comparison | Mean Difference | \|q\| | P | 95% CL |
| Wild type vs SAB-3mNG/LA | 0.244696 | 3.7505 | 0.0894 | -0.020544 to 0.50994 |
| Wild type vs mCh-Tub | 0.102577 | 1.5665 | 0.8778 | -0.16362 to 0.36878 |
| Wild type vs SAB-3mNG/Tub | 0.0808652 | 1.2205 | 0.9548 | -0.18849 to 0.35022 |
| Wild type vs LAmRuby | 0.0740657 | 1.1225 | 0.9684 | -0.19419 to 0.34232 |
| Wild type vs SAB-3GFP | 0.0187581 | 0.2843 | 1 | -0.2495 to 0.28701 |
| SAB-3GFP vs SAB-3mNG/LA | 0.225938 | 4.3024 | 0.0311 | 0.012446 to 0.43943 |
| SAB-3GFP vs mCh-Tub | 0.0838188 | 1.5872 | 0.8717 | -0.13087 to 0.2985 |
| SAB-3GFP vs SAB-3mNG/Tub | 0.0621071 | 1.1551 | 0.9642 | -0.15647 to 0.28069 |
| SAB-3GFP vs LAmRuby | 0.0553076 | 1.0351 | 0.9778 | -0.16192 to 0.27253 |
| LAmRuby vs SAB-3mNG/LA | 0.17063 | 3.2492 | 0.1994 | -0.042861 to 0.38412 |
| LAmRuby vs mCh-Tub | 0.0285113 | 0.5399 | 0.9989 | -0.18617 to 0.2432 |
| LAmRuby vs SAB-3mNG/Tub | 0.00679953 | 0.1265 | 1 | -0.21178 to 0.22538 |
| SAB-3mNG/Tub vs SAB-3mNG/LA | 0.163831 | 3.0997 | 0.2457 | -0.051038 to 0.3787 |
| SAB-3mNG/Tub vs mCh-Tub | 0.0217117 | 0.4085 | 0.9997 | -0.19434 to 0.23777 |
| mCh-Tub vs SAB-3mNG/LA | 0.142119 | 2.7395 | 0.3824 | -0.068787 to 0.35302 |

**Supplemental File 1h.**

One Way ANOVA for Figure 4 – figure supplement 3D, left graph

Analysis of Variance Results

| Source | DF | SS | MS | F | P |
| --- | --- | --- | --- | --- | --- |
| Total | 27 | 0.80545469 | 0.029831655 |  |  |
| A | 2 | 0.67424666 | 0.33712333 | 64.234506 | < .0001 |
| Error | 25 | 0.13120803 | 0.0052483213 |  |  |

| Tukey's All Pairs Comparison | | | | |
| --- | --- | --- | --- | --- |
| Comparison | Mean Difference | \|q\| | P | 95% CL |
| ER-mCherry vs mCherry-Tub | 0.36521 | 15.8655 | < .0001 | 0.28412 to 0.4463 |
| ER-mCherry vs LA-mRuby | 0.190222 | 7.9209 | < .0001 | 0.10563 to 0.27482 |
| LA-mRuby vs mCherry-Tub | 0.174988 | 6.6003 | 0.0003 | 0.081597 to 0.26838 |

**Supplemental File 1i.**

One Way ANOVA for Figure 4 – figure supplement 3D, right graph

Analysis of Variance Results

| Source | DF | SS | MS | F | P |
| --- | --- | --- | --- | --- | --- |
| Total | 31 | 0.70763955 | 0.022827082 |  |  |
| A | 2 | 0.46350005 | 0.23175002 | 27.528322 | < .0001 |
| Error | 29 | 0.2441395 | 0.0084186034 |  |  |

| Tukey's All Pairs Comparison | | | | |
| --- | --- | --- | --- | --- |
| Comparison | Mean Difference | \|q\| | P | 95% CL |
| ER-mCherry vs mCherry-Tub | 0.295124 | 10.1205 | < .0001 | 0.19328 to 0.39697 |
| ER-mCherry vs LA-mRuby | 0.198497 | 7.3295 | < .0001 | 0.10391 to 0.29308 |
| LA-mRuby vs mCherry-Tub | 0.0966266 | 3.3775 | 0.0596 | -0.0032935 to 0.19655 |
